# First feasibility demonstration of GNSS-seismology for anthropogenic earthquakes detection

**DOI:** 10.1038/s41598-023-47964-2

**Published:** 2023-11-27

**Authors:** Iwona Kudłacik, Jan Kapłon, Kamil Kazmierski, Marco Fortunato, Mattia Crespi

**Affiliations:** 1 Institute of Geodesy and Geoinformatics, Wrocław University of Environmental and Life Sciences, Grunwaldzka Str. 53, 50-357 Wrocław, Poland; 2 Geodesy and Geomatics Division, DICEA-Sapienza University of Rome, Via Eudossiana 18, 00184 Rome, Italy

**Keywords:** Natural hazards, Environmental sciences

## Abstract

High-rate GNSS has been proven effective in characterising waveforms and co-seismic displacements due to medium-to-strong natural earthquakes. No application focused on small magnitude events like shallow anthropogenic earthquakes, where displacements and noise have the same order of magnitude. We propose a procedure based on proper signal detection and filtering of the position and velocity time series obtained from high-rate (10 Hz) GNSS data processing with two intrinsically different approaches (Precise Point Positioning and variometry). We tested it on five mining tremors with magnitudes of 3.4–4.0, looking both at event detection and its kinematic characterisation. Here we show a high agreement, at the level of 1 s, between GNSS and seismic solutions for the earthquake first epoch detection. Also, we show that high-rate multi-constellation (GPS + Galileo) GNSS can reliably characterise low-magnitude shallow earthquakes in terms of induced displacements and velocities, and, including their peak values, respectively, at the level of very few millimetres and 1–2 cm/s, paving the way to the routine use of GNSS-seismology for monitoring human activities prone to cause small earthquakes and related potential damages.

## Introduction

High-rate GNSS (HR-GNSS) dynamic monitoring has been applied to seismology (ground motion induced by natural earthquakes and their magnitude estimation, generally with M > 5)^1–12^ and structural health monitoring (generally bridges, towers, and slender structures)^13–21^. Although the sampling rate of HR-GNSS is lower than seismic records, it is generally sufficient to capture the waveforms correctly, as evidenced by the high agreement with the seismic records, considering the Nyquist frequency^18,22,23^. On the other hand, it is well known that accelerometers (or velocimeters) can, in principle, supply all these quantities with higher accuracy and at a much higher time resolution than GNSS (observation rate up to hundreds of Hz). However, they do not guarantee the representation of the overall event in a unique reference frame (each sensor has its internal reference frame) and may suffer from the so-called baseline shift (acceleration bias). Additionally, the integration of acceleration to obtain displacement and velocity introduces low-frequency noise, which is commonly reduced with high-pass filters^24^. With HR-GNSS, displacements or velocities are directly obtained with proper observation processing, e.g., Precise Point Positioning (PPP)^2,25^ or variometric approach (VAD, based on VADASE)^3,26^, respectively.

A much less addressed application of HR-GNSS, which sounds like a bridge between seismology and structural health monitoring, is monitoring anthropogenic earthquakes (e.g., due to underground excavations or mining activities)^22,27,28^. The mining tremors generally have smaller magnitude (M <  = 4.5) but also much shallower epicentres (depths up to 1–2 kms) than natural earthquakes, so they can dangerously impact the ground, structures, and infrastructures nearby, even if the waveform amplitudes are small, within the level of few centimetres. The small anthropogenic earthquakes usually do not induce sudden permanent coseismic displacements, but in mining areas, displacements related to mining-induced subsidence are observed^29^. GNSS applications for monitoring mining areas are closely linked to the protection of built-up areas, infrastructure, and the environment against landslides and subsidence.

To meet the application needs, it is necessary, at first, to reliably detect these anthropogenic events, to estimate the induced accelerations, velocities and displacements, and the dominant frequencies. These parameters are essential from the structural engineering and applied geology viewpoint to judge on the possible damages^30,31^. The HR-GNSS usually supply displacements and velocities in terms of their peak values, but the peak values of accelerations (PGA) were considered too noisy to reliably retrieve. However, recently a novel method to reconstruct the HR-GNSS velocity and acceleration with significantly reduced noise was proposed and assessed on the example of strong natural earthquake^32^.

Strong natural earthquakes are nowadays commonly monitored with HR-GNSS, and the observations are included into early warning systems; part of which are the earthquake detection procedures. The procedures for detecting earthquakes usually assume an a priori threshold of displacement, above which an event is identified, and they often rely on the seismic algorithm to detect ground motions associated with sudden ground deformations named STA/LTA (short-term data average versus long-term data average trigger algorithm)^33–35^. However, in case of small-magnitude earthquakes where the vibration amplitudes are at the level of noise, the proper, careful denoising approach, as well as less constrained event detection procedure, are needed, which is a subject of this study.

Here we show that GNSS-seismology can be successfully extended to anthropogenic earthquakes, meeting all these needs, provided: (i) approaches for absolute displacements (PPP) and velocities (VAD) estimation, which can run independently in parallel on the same GNSS data and be used together, benefiting of their best features^1–9^; (ii) a proper signal detection and filtering is adopted to extract the relevant kinematic information with the due accuracy from the background noise.

## Results

Hereafter we discuss the application of our procedure to short-term waveforms caused by anthropogenic earthquakes with magnitudes 3.4–4.0 due to mining activity (mining tremors) in two areas with different ground characteristics. The application of HR-GNSS was first demonstrated in recent years, showing that if properly filtered (i.e. band-pass filtering), it can achieve a high correlation with seismic records^22,36^. In the present study, the filtering methodology is here reinforced with a more objective algorithm; also the first motion detection is presented here.

It has to be recalled that the capability of detecting waveforms at sub-centimetre level with high-rate PPP was proved during a synthetic experiment by Xu et al.^22^, but this has never been confirmed on data recorded during a real seismic event.

In this study, we considered five mining tremors that occurred in Poland – the details are presented in Supplementary Materials (Supplementary Fig. 1 and Supplementary Table 1). The tremors are localized in two mining regions—Legnica-Głogów Copper District (LGOM) and Upper Silesian Coal Basin (GZW). Both areas are characterized by the complex geological structure that encumbers seismic wave propagation due to local anomalies; the tremors are often and strictly connected to mining activity.

The analysed HR-GNSS data has a 10 Hz sampling frequency; as reference, we used the data recorded with co-located strong motion stations (SM), which were available for three events. Two types of GNSS antenna-receiver sets were used: LEIAR20 LEIM—LEICA GR30 on CES1 and LES1 stations and TRM57971.00 NONE—TRIMBLE NETR9E on TRZB and TARN stations.

### Quality of the high-rate GNSS time series

The quality (accuracy) of the PPP and VAD time series close to the investigated events but in an undisturbed situation (120 s before each event) for both single (GPS – G) and multi (GPS + Galileo – G + E) constellation solutions was preliminarily assessed. The accuracy has been evaluated using the mean absolute error (MAE), used later to compare PPP and VAD time series in between and with the time series coming from seismometers (SM), where available. MAE has been recognised as a more robust and appropriate index than the standard root mean square error for comparing different methodologies measuring the same phenomenon and the related evaluation of the average discrepancy^37^. Therefore, using MAE for accuracy evaluation allows straightforward comparisons of the accuracies with the average discrepancies for all the considered time series. Overall results for the undisturbed uncorrected time series are presented in Table 1 with reference to the native products of the two approaches (displacements for PPP and velocities for VAD): the use of the G + E solution significantly decreases the MAE—for VAD-velocity by about 30% and for PPP-displacement by about 60%. Since the G + E solutions have significantly higher accuracy, only these solutions were considered in the further analysis.

**Table 1:** The MAE for the VAD-velocity and PPP-displacement for GPS-only (G) and GPS + Galileo (G + E) solutions, distinguishing ENU components and the full horizontal component (HZ).

GNSS constellation	MAE – VAD-velocity (mm/s)				MAE – PPP-displacement (mm)			
	E	N	U	HZ	E	N	U	HZ
	Uncorrected time series							
G	7.3	12.3	17.1	15.2	10.8	12.6	25.7	19.0
G + E	5.0	8.7	12.4	10.9	3.1	5.6	10.8	7.4
	Filtered time series							
G	2.9	5.2	5.0	6.4	0.6	1.0	2.0	1.3
G + E	**2.0**	**3.0**	**2.6**	**3.9**	**0.5**	**0.9**	**1.1**	**1.3**

Values for G + E solution, used in further study, are in bold.

Two upper rows refer to uncorrected time series, two lower rows to filtered time series. The accuracy was calculated using the 120 s period.

Nevertheless, also for G + E solutions, the native HR-GNSS time series (hereafter named “uncorrected”) are impacted by low- and high-frequency errors—fluctuations due to model errors—e.g. satellite orbits and clocks errors, and noise, respectively. The accuracy of HR-GNSS for short periods (few minutes) reaches the level of a few millimetres in the horizontal components^7,9,32^. The positioning accuracy is then comparable (if not higher) to the size of the events we aim to monitor. To detect and characterise the kinematics of these small vibrations, it is necessary to mitigate the impact of noise at high and low frequencies as much as possible.

To this aim, a procedure is here performed based on the Multiresolution Analysis—Interquartile Range (MRA-IQR) method described in the Methods section. In general, with MRA-IQR, the seismic signal is decomposed into multiple levels of various frequencies. We then analyze these levels to identify and select those containing co-seismic vibrations, which are later included into reconstructed signal. For small-magnitude anthropogenic earthquakes, the permanent co-seismic displacement is typically absent. Therefore, we exclude the low-frequency decomposition levels during our analysis (Tables 3, 4). However, in the case of natural earthquakes where co-seismic displacement is more common, the low-frequency decomposition levels should be examined to capture this displacement.

Our accuracy analyses show that proper filtering of low-frequency fluctuations and high-frequency noise can reduce the MAE by more than 60% for VAD-velocities and 80% for PPP-displacements to the level of 2–3 mm/s and 0.5–1.0 mm in the East, North, Up components (E, N, U, respectively), as presented in Table 1. These preliminary results highlight the great potential to utilise the multi-GNSS solution for small vibrations analysis and lead to characterising the kinematics of low-magnitude earthquakes with HR-GNSS time series.

### Tremors detection and duration

At first, the defined filtering technique, applied to the intrinsically less noisy horizontal components of the time series, leads to a high agreement of mining tremor first motion detection across the different sensors.

PPP and VAD detection of first motion agrees within 1.0 s MAE. In addition, also considering that the GNSS receivers and SM are not perfectly co-located in all sites (in TARN, the inter distance is about 200 m), the GNSS (mean value between PPP and VADASE) and SM detection of first motion agrees within 1.7 s MAE.

**Figure 1:**
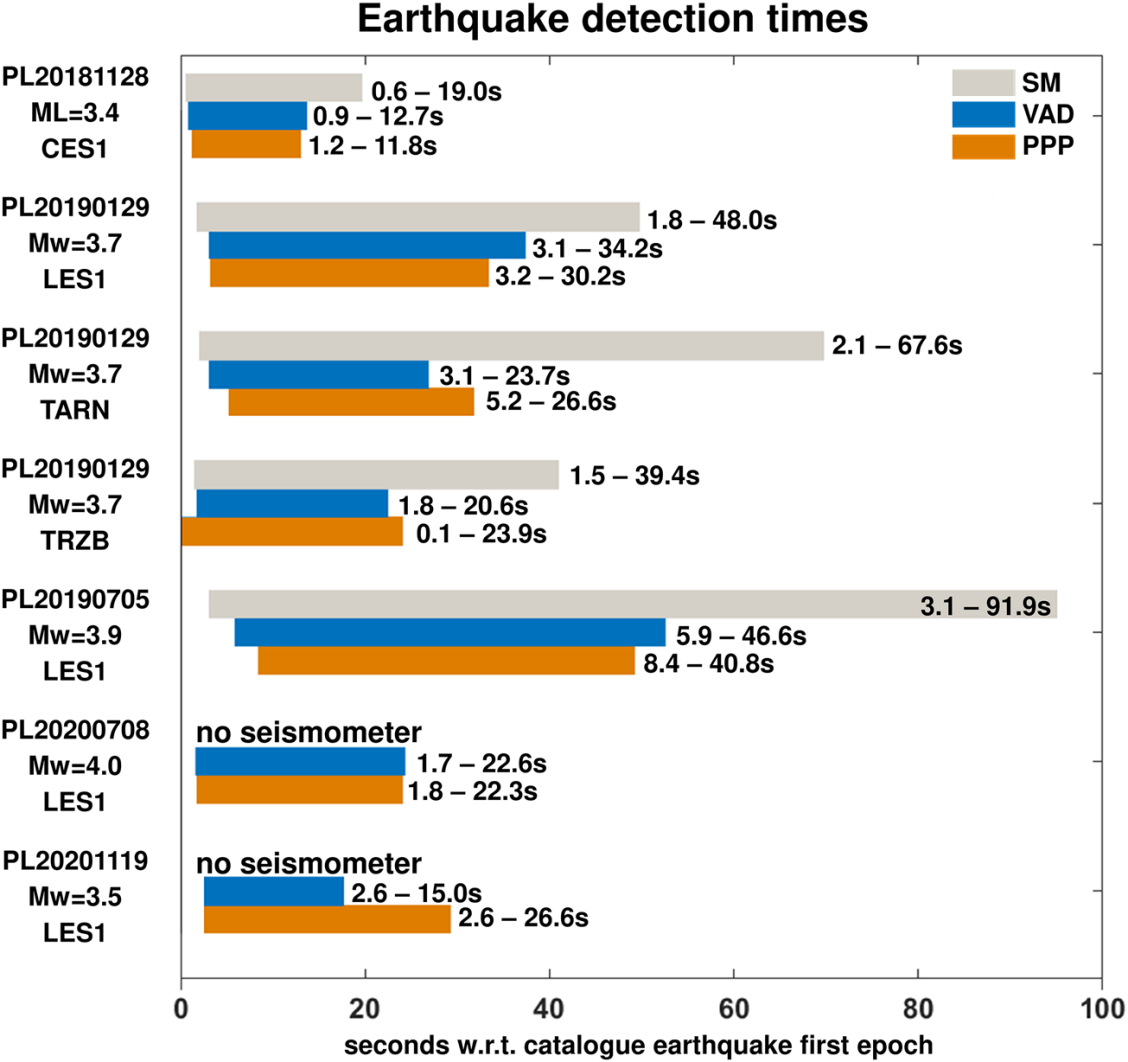
Epoch of detection of the tremor start and end depending on the technique (0 on the horizontal axis is the earthquake moment according to the seismic catalogue). For PPP, the analysis was conducted for displacements, for VADASE—for velocity, and for seismometers—for accelerations. Note, that TARN and TRZB have nonzero distance between GNSS and seismic station. *Mw* denotes Moment magnitude scale, and *ML* is local magnitude—this depends on seismic catalogue.

However, there are differences in the end-of-event detection and, therefore, in the estimation of earthquake duration, mainly due to the much higher sensitivity of seismometers with reference to GNSS solutions. SM are able to detect very small vibrations also when they are well below the level of GNSS time series noise for both PPP and VAD approaches; therefore, tremor durations are usually longer when estimated from SM data (Fig. 1, panel B).

Additionally, the normality analysis of the horizontal components of the time series based on the Jarque–Bera test, as presented in Methods subsection—“Normality Tests”, highlighted that tremors act as outliers with reference to the normality of the time series, and they significantly impact the Jarque–Bera test statistic; therefore anthropogenic earthquakes can be noticed looking at this test statistic (Fig. 5).

### Tremor kinematic features

Three approaches (SM, PPP, VAD) were used to determine the size of vibrations during tremors and their kinematic features in terms of displacements, velocities and accelerations. The accuracy (MAE) of the GNSS (PPP & VAD) corrected displacements, velocities and accelerations are at the level of 0.7 mm, 3.3 mm/s, and 2.1 cm/s^2^. The details are presented in Table 2.

**Table 2:** The MAE for the filtered VAD and PPP time series: displacements, velocity, and accelerations distinguishing ENU components and the full horizontal component (HZ).

Time series	VAD				PPP			
	E	N	U	HZ	E	N	U	HZ
Displacements (mm)	0.4	0.7	0.8	0.8	0.5	0.9	1.1	1.3
Velocity (mm/s)	2.0	3.0	2.6	3.9	3.8	3.7	5.0	5.9
Acceleration (mm/s^2^)	20.8	31.1	13.3	40.1	22.8	21.3	27.9	34.4

The maximum size of the vibrations is represented using peak ground displacement (PGD), peak ground velocity (PGV), and peak ground acceleration (PGA); PGD and PGA are relevant to represent the earthquake impact from the geometric and the dynamic point of view. The agreement among the analysed approaches is represented in terms of MAE and Pearson’s correlation coefficient of the time series. Detailed information about the calculation of these quantities is in the Methods section.

The median correlation coefficient value for SM/GNSS horizontal displacements is 0.77, and for vertical – 0.64. This value calculated for velocities reaches 0.83 and 0.58, respectively. The agreement between SM and GNSS acceleration observations is slightly worse, at the level of 0.62 and 0.50, respectively, due to high noise values. The correlation coefficient values are summarised in Table 5 in the Methods section.

**Figure 2:**
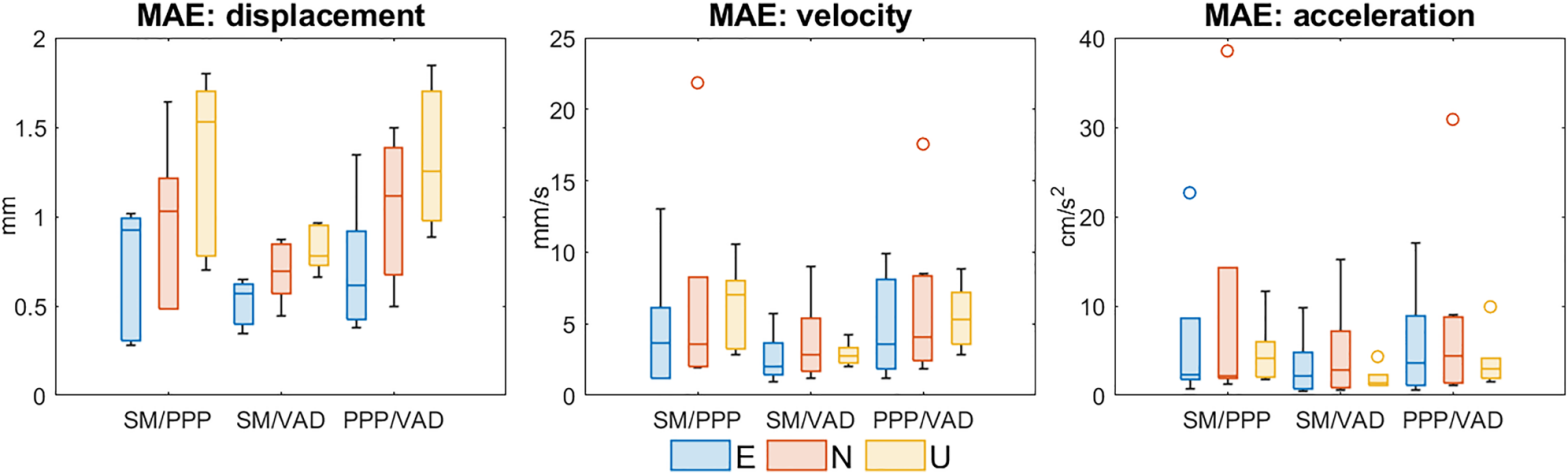
Summary statistics with boxplots of mean absolute error (MAE) between three approaches: SM, PPP, and VAD. Note that the panels have different scales. Each boxplot displays following information: median, the lower and upper quartile, maximum and minimum value and outliers, if present (denoted with the circles).

PGD median values for all the considered events were in the range of 5–10 mm, with an increasing dispersion (interquartile Q3-Q1 range) moving from PPP to VAD and SM. The detailed PGD values are presented in Supplementary Table 2. MAE values for displacements confirm a very good VAD-SM agreement well within 0.5–1 mm, and good PPP-VAD and PPP-SM agreements within 1–2 mm, slightly worse for the Up component (Fig. 2).

However, it is important to note that one event (CES1 - 2018–11–28) is located in an area where very high-frequency vibrations are induced by the earthquake, resulting in a lower agreement between GNSS and SM time series. Seismometers were able to capture these vibrations due to their higher sensitivity and acquisition rate. In contrast, GNSS results are impacted by aliasing, and the correlation between GNSS and SM displacements and velocities in the case of CES1 is poor (Supplementary Fig. 2). Nevertheless, even in this situation, it is possible to correctly detect the earthquake with GNSS, as it is shown in Fig. 1.

PGV median values for all the considered events were in the range of 15–35 mm/s, with a decreasing dispersion (interquartile Q3-Q1 range) moving from PPP to VAD and SM. The detailed PGV values are presented in Supplementary Table 2. MAE values for velocities confirm a very good VAD-SM agreement well within 5 mm/s and good PPP-VAD and PPP-SM agreements within 10 mm/s, slightly worse for the Up component (Fig. 2).

## Conclusions

We demonstrated that multi-constellation GNSS (GPS + Galileo) is able to detect and retrieve the kinematic features of the waveforms due to small magnitude anthropogenic earthquakes, with results well in agreement with those supplied by seismometers, provided proper processing approaches and filtering are applied.

As regards processing, the presented results from HR-GNSS data (displacements, velocities and accelerations) were obtained with two independent post-processing approaches (PPP and VAD), which can run independently in parallel on the same GNSS data and which directly provide respectively displacements and velocities as native estimation products. The proposed filtering procedure (Multiresolution Analysis—Interquartile Range) was successful in highlighting the small signal related to the investigated earthquakes within the HR-GNSS time series, suppressing low-frequency fluctuations not related to earthquakes and high-frequency noise. This procedure led to obtaining the accuracy of very few millimetres for displacements and 1–2 cm/s for velocities, representing satisfactory results when compared with those retrieved from SM. Regarding the accelerations, those derived from VAD-velocities display a better (even if not yet satisfactory) agreement with those derived from SM, likely because one finite differentiation is involved only.

We conducted a series of tests on several mining tremors of magnitudes in the range of 3.4–4.0, and for the first time, we verified that it is possible to achieve on real data the same accuracy level previously obtained in tests under controlled laboratory conditions. It is worth underlining that to the best of our knowledge, magnitude 3.4 is the smallest detected with 10 Hz HR-GNSS data for either natural or anthropogenic earthquakes.

The presented results raise conclusions that a dense HR-GNSS network would be able to detect small anthropogenic earthquakes and estimate the induced displacements and accelerations, effectively complementing (and maybe replacing, in future) much higher-cost seismic stations. Possibly, in future, this could be even realised wit low-cost dual frequency receivers, however this needs further investigation, including improvements in noise reduction. An additional side advantage of HR-GNSS vs accelerometers (velocimeters) is the possibility of continuous long-term monitoring in parallel to dynamic monitoring. However, it is necessary to note that the GNSS-retrieved accelerations are characterised by a huge high-frequency noise, which results in mediocre correlations with SM. The first methodology to retrieve reliable GNSS-accelerations was recently presented by Xu et al.^32^ for the case of strong natural earthquake. We think finding the source and solution for reducing the high-frequency noise for moderate and small vibrations should be the subject of further research to retrieve reliable GNSS-accelerations.

The herein presented tests were conducted in post-processing. Therefore, further studies should consider, if the proposed procedure for earthquake detection and estimation of the induced displacements and accelerations could be implemented in (quasi) real-time, since both VAD and also PPP (provided is kept initialised). Also, in future, the HR GNSS could satisfy the additional need to implement a simple and cheap monitoring system, able to manage a potentially high and variable number of monitoring sites, to support also long-term monitoring against slow displacements, and to overcome the mentioned issues regarding accelerometers (and velocimeters).

## Methods

**Figure 3:**
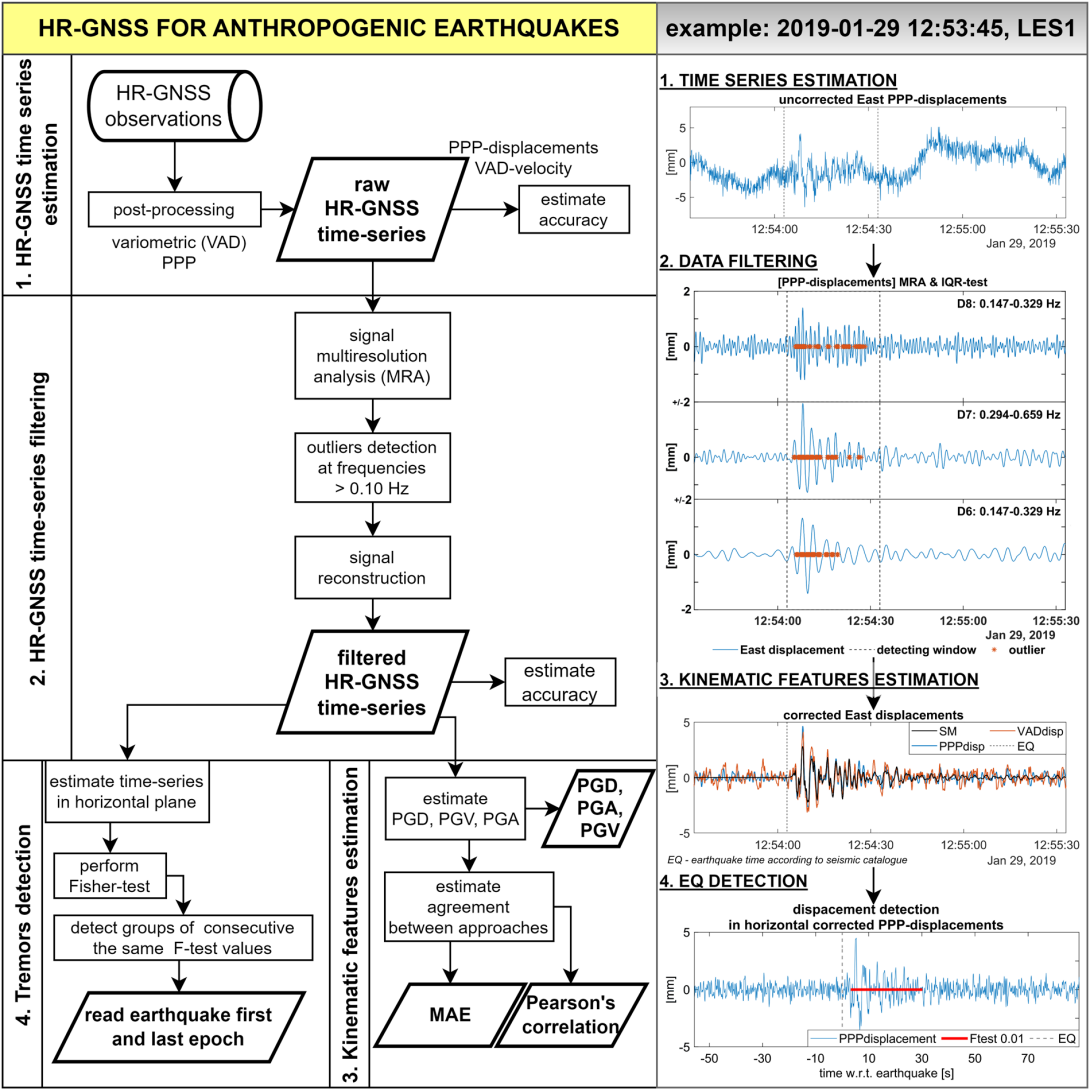
The logical flowchart of presented approach (left panel) with the example intermediate results of the main steps (right panel).

The approach hereafter illustrated is a bandpass filtering procedure (Multiresolution Analysis—Interquartile Range) which applies to the HR-GNSS time series (10 Hz observation rate) derived from Precise Point Positioning (PPP) and Variometric approach (VAD); it aims is to highlight the small signal induced by the anthropogenic earthquakes, suppressing both low-frequency fluctuations and high-frequency noise. The procedure flowchart is presented in Fig. 3, while the details are described in the following subsections.

### GNSS observations processing

The positions obtained with the PPP approach were calculated using GNSS-Wroclaw Algorithms for Real-Time Positioning software (GNSS-WARP)^38^, using the final IGS repro3 products from Graz University of Technology (TUG)^39^. In this software, the functional model of PPP processing uses the undifferenced uncombined approach^40^ without ambiguity resolution. For calculations, 6 h of observations were taken with mixed observations interval, in detail: first 5 h at 60 s acquisition rate, then 30–45 min at 10 Hz acquisition frequency and then 30–45 min again at 60 s acquisition rate. The 5 h period at the beginning of the computation ensures proper PPP initialisation. The difference in 10 Hz acquisition frequency duration depends on the time location of the interval of the tremor in the 15-min file. The final positions were converted to the displacements (PPP-displacements) with respect to the initial position of the GNSS station.

The velocities obtained with the Variometric approach were calculated with the VADASE software (Variometric Approach for Displacement Analysis Standalone Engine) developed by Colosimo et al.^41^, using the broadcast IGS orbits and only 15-min observation RINEX file. The processing results were epoch-by-epoch displacements, equivalent to velocities (here denoted as VAD-velocities).

For the developed analysis, we considered time series 245 s long, i.e., 120 s before and 95 s after each earthquake; the starting time of each earthquake has been taken from a seismological catalogue. The seismological data from the EPOS-PL seismic catalogue^42^ and the Central Mining Institute, Poland (GIG) were used to validate the results of geodetic techniques.

### GNSS-position changes filtering

Since the HR-GNSS time series suffers from low-frequency fluctuations and high-frequency noise, it is necessary to suppress both of them to retrieve and analyse the small seismic signal due to the considered small earthquakes.

**Figure 4:**
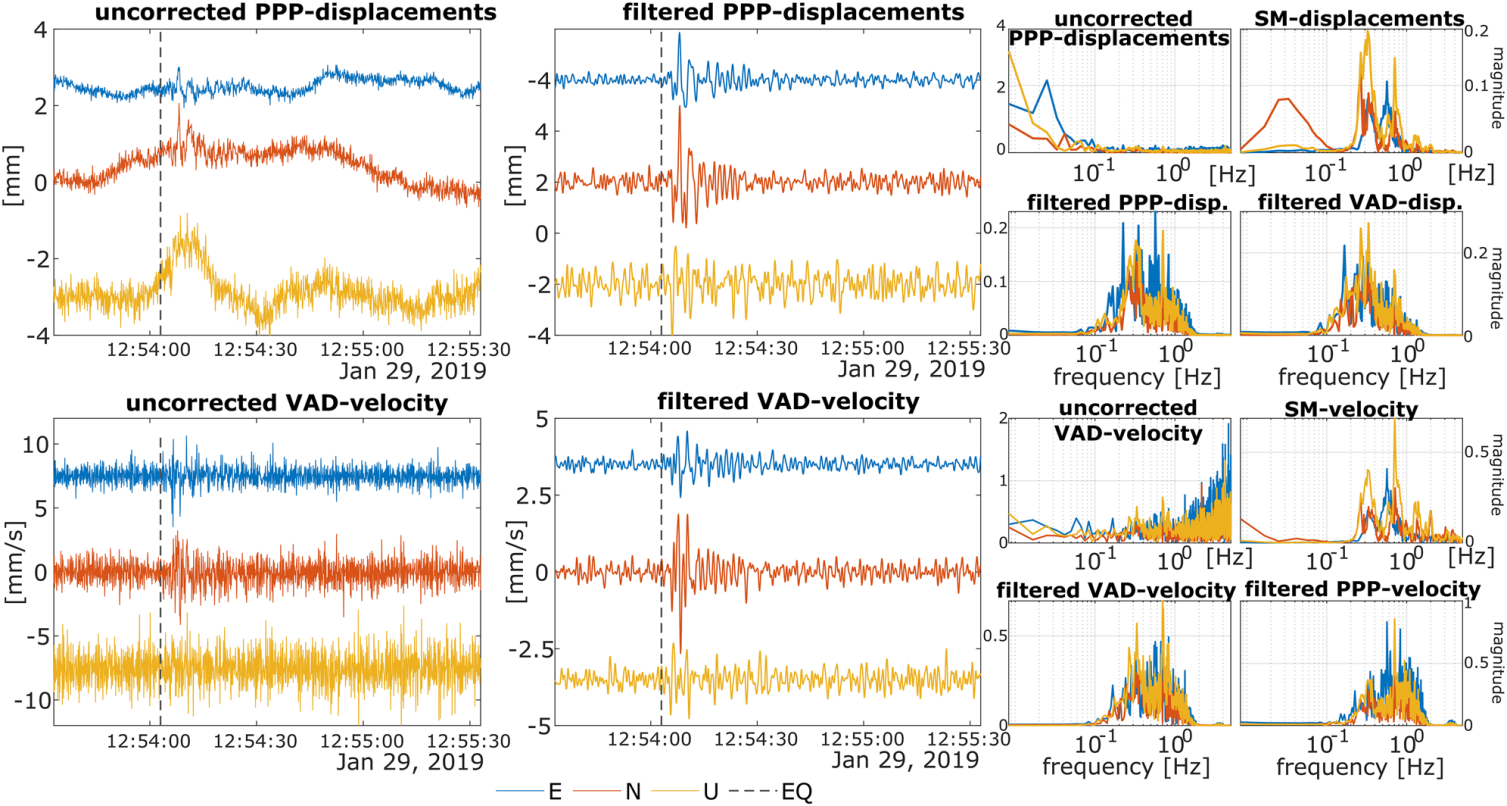
An example of VAD-velocity and PPP-displacement filtration results using MRA-IQR. Column 1 shows uncorrected time series, impacted by low-frequency fluctuations and high-frequency noise. Column 2 shows time series after MRA reconstruction. The dotted vertical line marks the earthquake starting epoch according to the seismic catalogue (EQ). Columns 3–4 show the frequency spectra of raw and corrected GNSS time series (PPP and VAD) with reference to seismic data (SM). In columns 1–2 the East, North and Up time series were shifted vertically for clarity.

We propose a three-step wavelet-based denoising method for the filtration of the HR-GNSS time series. In the first step, the signal decomposition is performed with multiresolution analysis (MRA)^43^. In the second step, the proper levels for reconstruction are identified with the interquartile-range test (IQR-test) with a level-dependent threshold. The last step is reconstructing the corrected (filtered) HR-GNSS time series. Figure 4 presents the results of applying that correction procedure for the 2019–01–29 earthquake. As it is clear, the filtration scheme keeps the main features of the earthquake signal, removing most of the undesired low-frequency fluctuations and high-frequency noise.

With the MRA, the signal is segmented with the selected wavelet and a series of low-pass and high-pass filters to build the pyramidal structure, resulting in the signal decomposition into various resolution levels. Here the signal is decomposed with the Daubechies 3 wavelet (db3) into 10 levels (D10–D1) and the Approximation level (trend component, denoted A). The Approximation level (A) corresponds to the lowest frequency components of the signal, while the D1–D10 levels encompass different frequency contents in ascending order. The D1 level represents the lowest frequency details, while the D10 level contains the highest frequency details among the decomposition levels. The frequency content of each decomposition level is in the heading of Tables 3 and 4. In the subsequent step, each decomposition level is examined to identify any signal perturbations present.

**Table 3:** List of MRA-IQR decomposition levels for PPP-displacements with indication of whether a tremor was identified, and the level selected for reconstruction.

Tremor UTC	GNSS	D10	D9	D8	D7	D6	D5	D4-A
		2.5–5.0 Hz	1.18–2.66 Hz	0.588–1.32 Hz	0.294–0.659 Hz	0.147–0.329 Hz	0.0737–0.165 Hz	0 -0.0825 Hz
		PPP	PPP	PPP	PPP	PPP	PPP	PPP
2018–11–28 11:35:24	CES1	**[HZ]**	■	■	■	■	□	□
2019–01–29 12:53:45	LES1	□	□	■	■	■	□	□
2019–01–29 12:53:45	TARN	□	□	■	■	■	□	□
2019–01–29 12:53:45	TRZB	□	□	■	■	■	**[HZ]**	□
2019–07–05 18:41:00	LES1	□	□	■	■	□	□	□
2020–07–08 05:19:00	LES1	□	**[HZ]**	■	■	■	□	□
2020–11–19 09:27:00	LES1	□	**[HZ]**	■	■	■	**[HZ]**	□

Marks: □- no outliers, [HZ]—only outliers in the horizontal directions, ■—outliers in all directions. The designation D4-A determines the joint D4-D3-D2-D1 levels and the Approximation (no outliers found here).

**Table 4:** List of MRA-IQR decomposition levels for VAD-velocities with indication of whether a tremor was identified, and the level selected for reconstruction.

Tremor UTC	GNSS	D10	D9	D8	D7	D6	D5	D4-A
		2.5–5.0 Hz	1.18–2.66 Hz	0.588–1.32 Hz	0.294–0.659 Hz	0.147–0.329 Hz	0.0737–0.165 Hz	0 -0.0825 Hz
		VAD	VAD	VAD	VAD	VAD	VAD	VAD
2018–11–28 11:35:24	CES1	**[HZ]**	■	■	■	□	□	□
2019–01–29 12:53:45	LES1	□	**[HZ]**	■	■	■	□	□
2019–01–29 12:53:45	TARN	□	□	■	■	■	□	□
2019–01–29 12:53:45	TRZB	□	**[HZ]**	■	■	■	**[HZ]**	□
2019–07–05 18:41:00	LES1	□	□	■	■	■	□	□
2020–07–08 05:19:00	LES1	□	**[HZ]**	■	■	■	□	□
2020–11–19 09:27:00	LES1	□	**[HZ]**	■	■	■	□	□

Marks: □- no outliers, [HZ]—only outliers in the horizontal directions, ■- outliers in all directions. The designation D4-A determines the joint D4-D3-D2-D1 levels and the Approximation (no outliers found here).

For the identification of the levels containing the earthquake signal, we benefited from an outlier detection strategy. We were looking for significant PPP-displacements and VAD-velocities, that is, outliers with respect to the undisturbed conditions (zero displacements, zero velocities), for each decomposition level (D) and separately for horizontal (HZ) and vertical (V) components. The IQR-test method of detecting outliers was recognised as effective for reconstructing the seismic signal in the HR-GNSS results. The IQR-test is based on determining the difference between the third and the first quartiles—interquartile range (Eq. 1).

1 $$IQR = Q3 - Q1$$

2 $$outlier:\left\{ {\begin{array}{*{20}l} {value < Q1 - 1.5 \cdot IQR} \hfill \\ {or} \hfill \\ {value > Q3 + 1.5 \cdot IQR} \hfill \\ \end{array} } \right.$$

In our analysis, the outliers are analysed in D10-D5 levels (Tables 3, 4). Considering the standard frequencies of the vibrations induced by shallow earthquakes, the decomposition levels with very low frequencies, i.e., below 0.08 Hz, are automatically rejected. Subsequently, to reconstruct the corrected signal, there are selected decomposition levels with a significant number of outliers detected with the IQR-test in the 30-s window starting from the epoch of the recorded event. The window length was selected to capture the anticipated entire duration of the seismic vibrations caused by the small-magnitude earthquake. The reconstructed signal contains of the selected levels that are summed together, following the Eq. (3):

3 $$Reconstructed\; signal = \mathop \sum \limits_{i = 1}^{n} D_{i} ,$$

where $D_{i}$ represents the i-th detail level selected for reconstruction and n represents the number of detail levels chosen for reconstruction.

In all tested cases, the levels in the frequency range 0.147–1.320 Hz are by far dominant in terms of the number of outliers, which was confirmed by evaluating the frequency composition of the tremors on co-located seismometers. Since the vertical component is usually burdened with higher noise, sometimes no outlier is detected; in such circumstances, the vertical component was reconstructed with the levels within the dominant frequency spectra (i.e., D8–D7–D6). The detailed list of the decomposition levels included into the filtered time series is presented in Table 3 for PPP-displacements and in Table 4 for VAD-velocities.

The effectiveness of the filter used in terms of removing low-frequency noise is visible in Table 1—the MAE value significantly decreases, e.g., for horizontal component from the level of 7.4 mm for uncorrected PPP-displacements and 10.9 mm/s for uncorrected VAD-velocities, to 1.3 mm and 3.9 mm/s, respectively.

### Normality assessment

The assessment of the data normality is necessary for the F-test used for earthquake detection. Within the different normality tests, we used the most recent one proposed by Jarque–Bera (JB-test)^44^. We assessed the normality of filtered PPP-displacements and VAD-velocities for the undisturbed situation before the earthquake, during the earthquake, and in the relaxation phase after an earthquake. We showed that the tremor affects the time series as outliers and results in a temporary loss of normality.

**Figure 5:**
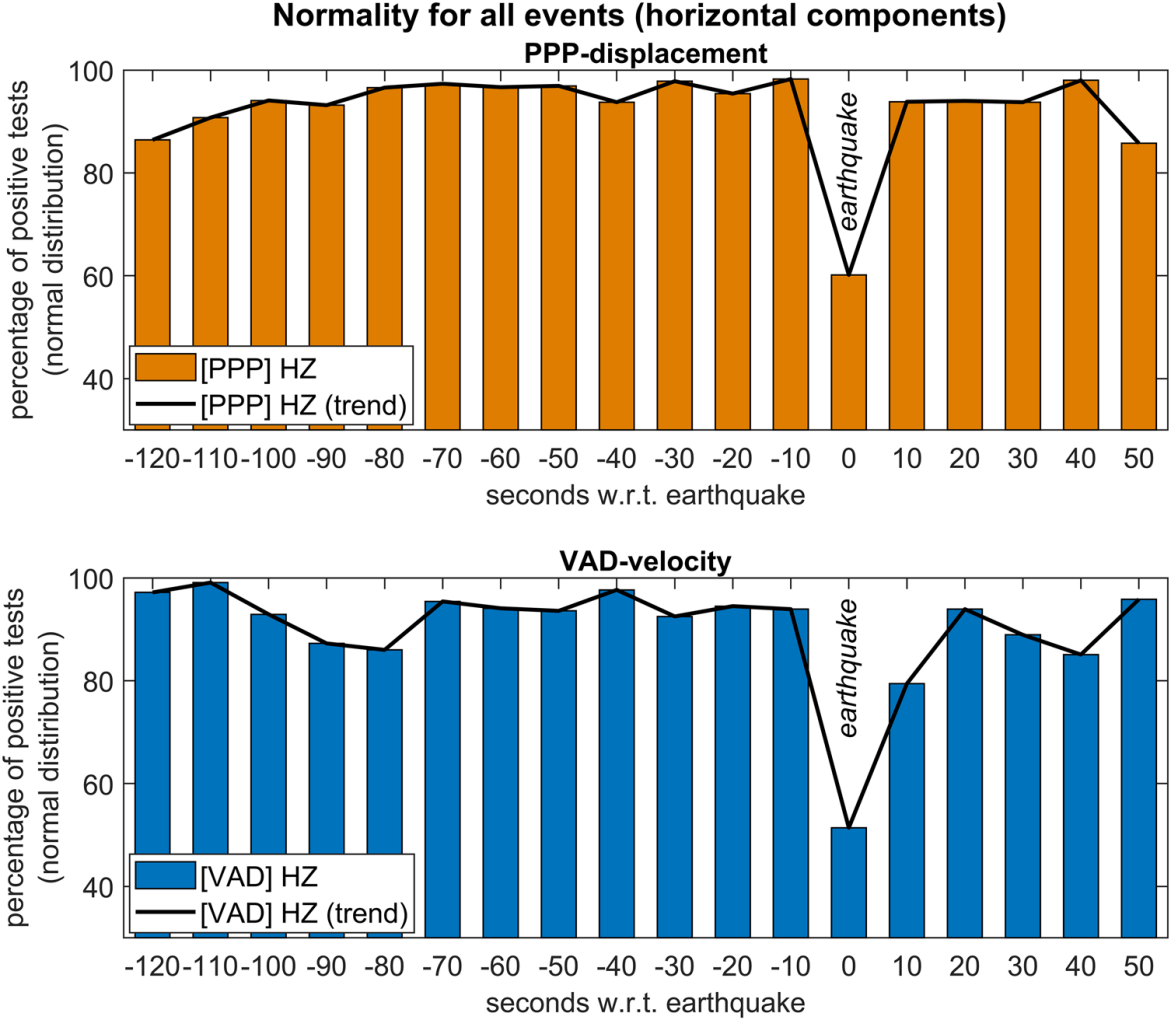
The percentage of normally distributed 10-s samples calculated for horizontal components of all analysed events, following the JB-test. The upper panel represents the normality for PPP-displacements, and the lower panel—the normality for VAD-velocity. With the red rectangle the period when the earthquake occurred is marked.

The normality was calculated with a 10 s moving window separately for East, North and Up components. For all events, there is a clear decrease in the number of positive tests for the period when the earthquake occurred. This means that the normality of time series during an event is temporally disturbed, as visible in Fig. 5—the upper panel presents time variability of the *p*-value (so-called observed significance level of the test) for PPP-displacements and VAD-velocities.

### Earthquake detection

The detection of tremor involves applying the Fisher test (F-test) to filtered time series data of displacements, velocities, or accelerations). The detection was performed on values provides directly by each processing approach—i.e. PPP, VAD and SM, respectively. The algorithm consists of three steps: calculation of standard deviation, F-test application and tremor identification.

As with previous analyses, tremor detection is performed within 120 s before up to 95 s after the event. During the research, it was found that it is reasonable to present the horizontal components: East and North, as a vector sum and to perform the detection on this combination (hereinafter referred to as "horizontal" or HZ). The HZ is calculated by taking the square root of the sum of the squares of the East and North components.

To identify the tremor, we follow these steps. Firstly, the STD values were determined by shifting the 10-s moving window every epoch (0.1 s). The STD in stable conditions was obtained using the last 120 s before an event (according to the seismic catalogue). For each window, the STD values are computed and then averaged, providing an estimation of the STD under stable conditions.

Once the STD value for the stable conditions is determined, the F-test is applied epoch by epoch at a confidence level of 99%. The F-test is conducted using the Eq. (4):

4 $$F_{i} = \frac{{STD_{i}^{2} }}{{STD_{stable}^{2} }},$$

Where $STD_{i}$ – represents the temporal STD calculated for each 10-s moving window (denoted by i), and $STD_{stable}$ – represents standard STD under stable conditions.

Finally, the consecutive groups of F-test values with the same sign (positive or negative) are identified, considering a minimum duration of 10 s. This helps minimalize false alarms caused by sudden spurious spikes that may appear and last only a few epochs, while the tremor period typically exceeds 10 s in the analysed cases.

### Peak values and correlations

**Figure 6:**
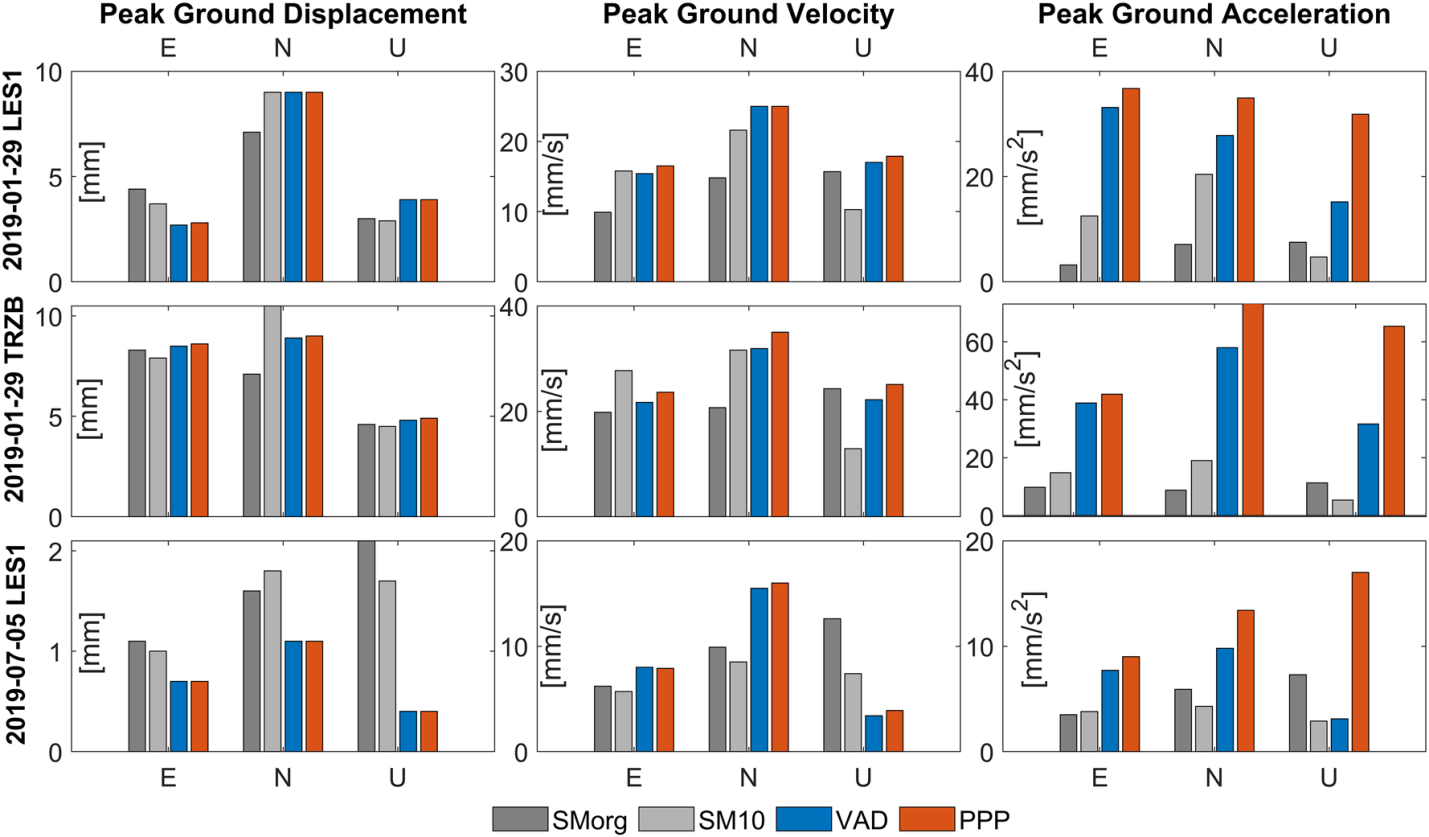
The values of PGD, PGV and PGA for three selected events calculated with GNSS and SM. SM10 denotes seismic records interpolated to 10 Hz sampling frequency, whereas SMorg denotes seismic records with original sampling frequency.

The maximum size of the vibrations during the earthquakes was characterised by the values of Peak Ground Displacement (PGD), Peak Ground Velocity (PGV), and Peak Ground Acceleration (PGA). These values are calculated as the maximum absolute values of the time series during an earthquake. They are listed in Supplementary Table 2 for all analysed events, and the four selected examples are presented in Fig. 6.

Pearson’s correlation value is calculated with the 10-s moving window, shifting every epoch, and the maximum reached for the strongest vibrations is presented in the manuscript. From the set of the obtained correlations observed during the earthquakes, in Table 5, we present their medians.

**Table 5:** The median correlations summarizing the reached correlations for all analysed earthquakes.

Median	Displacements			Velocity			Acceleration		
	E	N	U	E	N	U	E	N	U
PPP/SM	0.74	0.75	0.64	0.77	0.77	0.53	0.63	0.62	0.43
VAD/SM	0.79	0.84	0.82	0.84	0.89	0.84	0.74	0.55	0.58
PPP/VAD	0.89	0.85	0.68	0.85	0.84	0.73	0.78	0.75	0.76

### Supplementary Information

Supplementary Information.

## Data Availability

The seismological data is available from the repository of EPOS Thematic Core Service Anthropogenic Hazards (EPISODES platform) at https://tcs.ah-epos.eu/#episode:LGCD (last accessed in September 2022). The non-filtered HR-GNSS coordinate time series used in this study are available at https://doi.org/10.5281/zenodo.7505641. The HR-GNSS raw data will be shared on reasonable request to the corresponding author.
