# Supplementary material for: First feasibility demonstration of GNSS-seismology for anthropogenic earthquakes detection
Source: Sci Rep. 2023 Nov 27;13:20905. doi: 10.1038/s41598-023-47964-2 (PMC10684640; doi:10.1038/s41598-023-47964-2)
Supplement: Supplementary file 1 — Supplementary Information. [file 41598_2023_47964_MOESM1_ESM.pdf]

Supplementary Material

First feasibility demonstration of GNSS-seismology for anthropogenic earthquakes detection

Iwona Kudłacik[1], Jan Kapłon[1], Marco Fortunato[2], Kamil Kazmierski[1], Mattia Crespi[2]

[1] Wrocław University of Environmental and Life Sciences, Institute of Geodesy and Geoinformatics, Grunwaldzka Str. 53, 50-357 Wrocław, Poland

[2] Geodesy and Geomatics Division, DICEA-Sapienza University of Rome, via Eudossiana 18, Rome, 00184, Italy

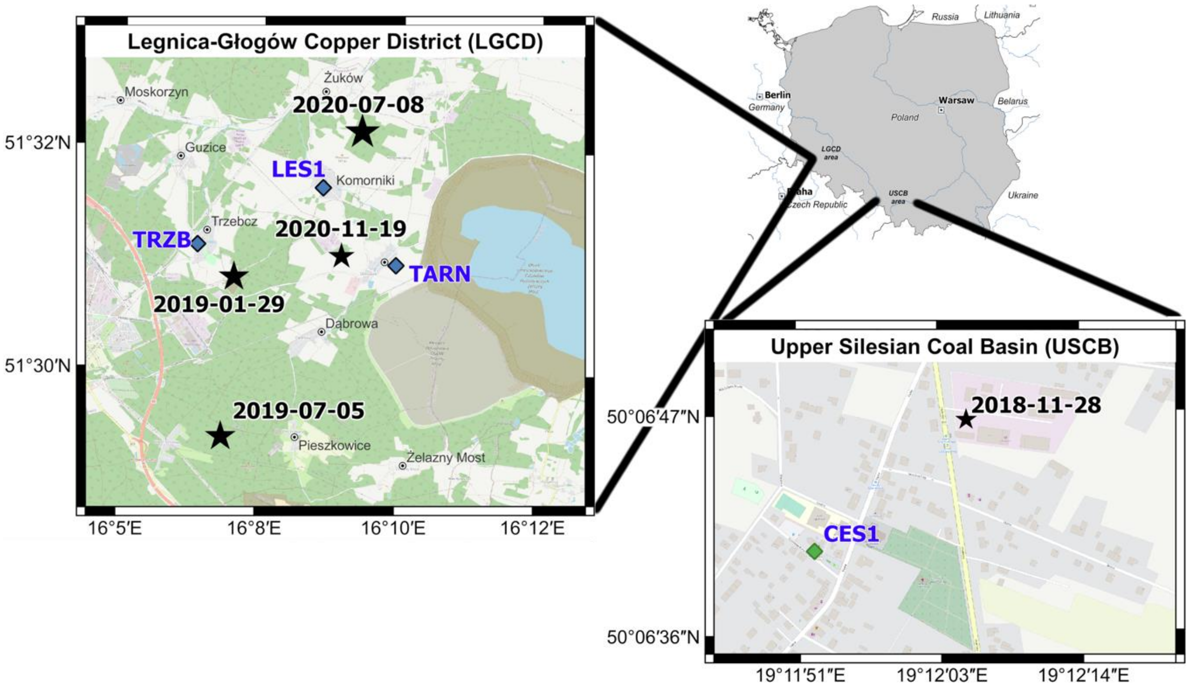

Supplementary Figure 1 Location of analysed earthquakes. The size of stars depends on event's magnitude. The maps were generated with QGIS ver.3.20 with the background from OpenStreetMap and Natural Earth dataset (https://www.naturalearthdata.com/).

Supplementary Table 1 Details of analysed earthquakes. Mw= Moment magnitude scale, ML=local magnitude – this depends on catalogue.

EARTHQUAKE [UTC]	MAGNITUDE	DEPTH [m]	GNSS STATION	STRONG-MOTION SENSOR	EPICENTRAL DISTANCE [km]
2018-11-28 11:35:24	ML=3.4	1000	CES1	CHELM	0.3
2019-01-29 12:53:45	Mw=3.7	800	LES1	KOMR	2.5
			TARN	TRN2	3.2
			TRZB	TRBC2	1.0
2019-07-05 18:41:00	Mw=3.9	800	LES1	KOMR	5.4
2020-07-08 05:19:00	Mw=4.0	2255	LES1	-	1.4
2020-11-19 09:27:00	Mw=3.5	800	LES1	-	1.4

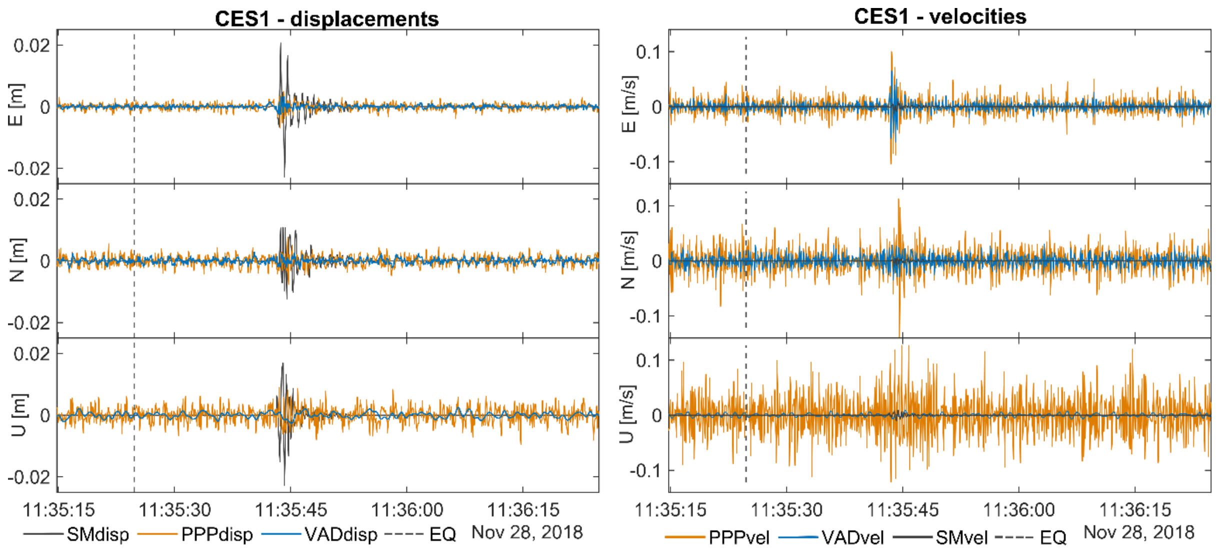

Supplementary Figure 2 The displacements and velocities of CES1 during event 2018-11-28 obtained with seismometer (SM) and two GNSS approaches: PPP and VAD. The dotted line marks the event moment, according to seismic catalogue (EQ).

Supplementary Table 2 List of Peak Ground Displacements (PGD), Peak Ground Velocities (PGV) and Peak Ground Accelerations (PGA) observed with PPP and VAD approaches, and seismic sensors. SM10 denotes seismic records interpolated to 10 Hz sampling frequency, whereas SMorg denotes seismic records with original sampling frequency.

	EARTHQUAKE	STATION	PPP			VAD			SM10			SMorg		
			E	N	U	E	N	U	E	N	U	E	N	U
PGD [mm]	2018-11-28	CES1	4.2	6.3	4.3	2.9	2.3	1.9	17.9	11.2	18.1	22.0	11.6	19.2
	2019-01-29	LES1	4.4	7.1	3.0	3.7	9.0	2.9	2.7	9.0	3.9	2.8	9.0	3.9
	2019-01-29	TARN	5.7	3.3	6.2	7.0	3.0	3.1	2.2	1.5	1.6	2.2	1.5	1.6
	2019-01-29	TRZB	8.3	7.1	4.6	7.9	10.5	4.5	8.5	8.9	4.8	8.6	9.0	4.9
	2019-07-05	LES1	1.1	1.6	2.1	1.0	1.8	1.7	0.7	1.1	0.4	0.7	1.1	0.4
	2020-07-08	LES1	4.4	15.4	5.7	4.8	17.1	4.4	x	x	x	x	x	x
	2020-11-19	LES1	3.0	2.0	2.0	2.9	2.3	2.8	x	x	x	x	x	x
PGV [mm/s]	2018-11-28	CES1	90.2	92.8	43.8	60.3	32.0	13.5	7.5	10.8	9.3	14.5	12.3	10.3
	2019-01-29	LES1	9.9	14.8	15.7	15.8	21.6	10.3	15.4	25.0	17.0	16.5	25.0	17.9
	2019-01-29	TARN	12.9	13.5	24.0	18.5	10.2	13.4	12.7	11.2	12.6	12.8	11.4	13.0
	2019-01-29	TRZB	19.8	20.7	24.3	27.7	31.6	12.9	21.7	31.9	22.2	23.6	35.0	25.1
	2019-07-05	LES1	6.2	9.9	12.6	5.7	8.5	7.4	8.0	15.5	3.4	7.9	16.0	3.9
	2020-07-08	LES1	30.8	53.2	30.6	24.8	57.0	19.5	x	x	x	x	x	x
	2020-11-19	LES1	14.7	18.3	10.1	10.8	18.6	10.4	x	x	x	x	x	x
PGA [cm/s^2]	2018-11-28	CES1	183.6	215.1	44.1	114.5	59.0	16.3	10.8	15.8	12.7	98.1	34.6	54.5
	2019-01-29	LES1	3.2	7.1	7.5	12.5	20.4	4.7	33.1	27.8	15.2	36.7	34.9	31.8
	2019-01-29	TARN	6.8	6.5	13.3	5.2	3.9	5.9	15.9	13.2	9.9	29.6	23.8	16.6
	2019-01-29	TRZB	9.8	8.8	11.3	14.8	19.0	5.4	38.8	57.9	31.6	41.9	73.4	65.3
	2019-07-05	LES1	3.5	5.9	7.3	3.8	4.3	2.9	7.7	9.8	3.1	9.0	13.4	17.0
	2020-07-08	LES1	29.2	50.9	16.0	24.1	52.9	11.4	x	x	x	x	x	x
	2020-11-19	LES1	19.9	15.6	5.9	11.3	13.2	4.0	x	x	x	x	x	x
